# Detection Efficacy of ^68^Ga-PSMA-11 PET/CT in Biochemical Recurrence of Prostate Cancer with Very Low PSA Levels: A 7-Year, Two-Center “Real-World” Experience

**DOI:** 10.3390/cancers15051376

**Published:** 2023-02-21

**Authors:** Caroline Burgard, Manuela A. Hoffmann, Madita Frei, Hans-Georg Buchholz, Fadi Khreish, Robert J. Marlowe, Mathias Schreckenberger, Samer Ezziddin, Florian Rosar

**Affiliations:** 1 Department of Nuclear Medicine, Saarland University—Medical Center, 66421 Homburg, Germany; 2 Department of Nuclear Medicine, Johannes Gutenberg University, 55101 Mainz, Germany; 3 Institute for Preventive Medicine Bw, 56626 Andernach, Germany; 4 Spencer-Fontayne Corporation, Jersey City, NJ 07304-1901, USA

**Keywords:** prostate cancer, biochemical recurrence, low serum prostate-specific antigen (PSA) concentration, gallium-68 prostate-specific membrane antigen-11 positron emission tomography (^68^Ga-PSMA-11 PET/CT), detection rate, PSA doubling time, Gleason score

## Abstract

**Simple Summary:**

Men with biochemical recurrence of prostate cancer (BCR) have increasing prostate-specific antigen (PSA) levels after potentially curative treatment, e.g., radical prostatectomy. Promptly finding these patients’ sites of recurrence potentially allows earlier, better-targeted treatment. Gallium-68 prostate-specific membrane antigen-11 positron emission tomography/computed tomography (^68^Ga-PSMA-11 PET/CT) imaging effectively localizes recurrence. However, this procedure’s detection rate of lesions suspicious for prostate cancer increases along with patients’ PSA levels, and limited published data describe performance when PSA is very low. We analyzed two academic institutions’ ~7-year “real-world” experience with ^68^Ga-PSMA-11 PET/CT in 115 prostatectomized men with BCR with very low PSA (≤0.2 ng/mL). Altogether, 44 suspicious lesions (median [minimum–maximum] 1 [1–4]/patient) were found in 29/115 men (25.2%). Multiple lesions were detected in 9/115 (7.8%) at PSA concentrations as low as 0.03 ng/mL. ^68^Ga-PSMA-11 PET/CT thus appears to be useful even in BCR with very low PSA.

**Abstract:**

In biochemical recurrence of prostate cancer (BCR), prompt tumor localization guides early treatment, potentially improving patient outcomes. Gallium-68 prostate-specific membrane antigen-11 positron emission tomography/computed tomography (^68^Ga-PSMA-11 PET/CT) detection rates of lesions suspicious for prostate cancer are well known to rise along with prostate-specific antigen (PSA) concentration. However, published data are limited regarding very low values (≤0.2 ng/mL). We retrospectively analyzed ~7-year “real-world” experience in this setting in a large post-prostatectomy cohort (N = 115) from two academic clinics. Altogether 44 lesions were detected in 29/115 men (25.2%) (median [minimum–maximum] 1 [1–4]/positive scan). The apparent oligometastatic disease was found in nine patients (7.8%) at PSA as low as 0.03 ng/mL. Scan positivity rates were highest when PSA was >0.15 ng/mL, PSA doubling time was ≤12 months, or the Gleason score was ≥7b (in 83 and 107 patients, respectively, with available data); these findings were statistically significant (*p* ≤ 0.04), except regarding PSA level (*p* = 0.07). Given the benefits of promptly localizing recurrence, our observations suggest the potential value of ^68^Ga-PSMA-11 PET/CT in the very low PSA BCR setting, especially in cases with more rapid PSA doubling time or with high-risk histology.

## 1. Introduction

Prostate-specific membrane antigen (PSMA), a transmembrane glycoprotein that is overexpressed on the surface of malignant prostate cells [1,2,3], has rapidly emerged as an important target in imaging [4,5,6] and treating this neoplasm [7,8,9]. In men with biochemical recurrence of prostate cancer (BCR), positron emission tomography/computed tomography (PET/CT) with radiotracers built on PSMA ligands is gaining growing use to try to localize tumor as accurately and early as possible, thereby attempting to optimize salvage therapy. As with PET/CT using other PSMA-targeted radiotracers [10,11,12], the detection rate with gallium-68 (^68^Ga)-PSMA-11 PET/CT of lesions suspicious for prostate cancer is well known to rise along with PSA concentration [13,14]. However, published data are relatively sparse regarding this imaging modality’s performance in men with BCR and very low (ultrasensitive) PSA concentrations, i.e., ≤0.2 ng/mL. To our knowledge, the literature regarding ^68^Ga-PSMA-11 PET/CT detection rates with this setting contains only two studies, including >100 patients with such very low PSA levels (those of Afshar-Oromieh et al. [15] and Meredith et al. [16]); additional reports covering this topic, e.g., [17,18,19,20,21,22,23,24,25,26,27], have involved much smaller samples.

Because the increasing use of PSMA-targeted PET/CT in prostate cancer is likely to be associated with greater use in earlier stages of BCR, it is important to characterize further ^68^Ga-PSMA-11 PET/CT performance in the very low PSA BCR setting. We, therefore, conducted the present analysis of our ~7-year “real-world” experience in a large dual-center cohort.

## 2. Patients and Methods

### 2.1. Study Design, Patients, and Endpoints

This was a retrospective analysis of ^68^Ga-PSMA-11 PET/CT findings in 115 consecutive men with a history of radical prostatectomy and with BCR marked by increasing but very low PSA concentration (≤0.2 ng/mL), as determined by local laboratories. Patients were referred by their treating urologist. Scanning took place between 15 January 2015 and 28 September 2022 at the outpatient nuclear medicine clinics of either of two German academic institutions, the Saarland University Medical Center, Homburg (*n* = 70, 60.9%) or the Johannes Gutenberg-University Medical Center, Mainz (*n* = 45, 39.1%).

The analysis had two main inclusion criteria. First, patients’ PSA concentrations had to be > 0 to ≤ 0.2 ng/mL. Second, patients had to have ^68^Ga-PSMA-11 PET/CT undertaken to seek structural correlates of PSA increase following primary treatment with radical prostatectomy.

Cohort characteristics are summarized in Table 1. The study sample tended to be in late middle age, and almost all had either an intermediate-risk (Gleason score = 7) or high-risk (Gleason score ≥ 8) histology of surgical samples (data available from 107/115 patients). At the time of scanning, the PSA concentration was <0.1 ng/mL, 0.1–0.15 ng/mL, or >0.15–0.2 ng/mL in about one-third of patients each. PSA doubling time was ≤12 months in more than four-fifths of patients with available data regarding this variable (*n* = 83/115; in the remaining patients, doubling time could not be calculated due to missing data regarding earlier PSA measurements). Only a small minority (<10% for either intervention) had received radiation or androgen deprivation therapy in addition to radical prostatectomy.

The primary endpoint of the analysis was the per-patient visual detection rate (positive scans/total scans) of any lesion(s) suspicious for prostate cancer. Secondary endpoints included the detection rate by lesion type (categorized as putative local recurrence, putative lymph node metastasis, or putative bone metastasis) and by PSA concentration, PSA doubling time, and Gleason score. Additional secondary endpoints included maximum standardized uptake values (SUV_max_) for suspicious lesions overall and by type.

The analysis was conducted according to the Declaration of Helsinki and approved by the Institutional Review Board of the Ärztekammer des Saarlandes/Saarbrücken (approval number: 170/22, approval date: 13 September 2022) and the ethics committee of the Medical Association Rhineland-Palatinate (Laek Rlp) (approval number: 2018/13390, approval date: 29 October 2018). All patients provided written informed consent for the ^68^Ga-PSMA-11 PET/CT and for the use of their de-identified data for scientific analyses and publications.

### 2.2. ^68^Ga–PSMA-11 PET/CT

All patients underwent whole-body scanning on ^68^Ga-PSMA-11 PET/CT ~60 min after intravenous injection (mean activity 157.8 ± 51.2 MBq) of the tracer followed by 0.9% NaCl, 500 mL; they were encouraged to void between the time of injection and that of data acquisition. PET/CT scans were performed on a Biograph 40 mCT PET/CT scanner (Siemens Healthineers, Erlangen, Germany; Homburg) or on a Gemini TF16 PET/CT scanner (Philips Medical Systems, Best, Netherlands; Mainz). The matrix was 200 × 200 (Homburg) or 168 × 168 (Mainz), the acquisition time was 3 min/bed position, the extended field-of-view was 21.4 cm (TrueV) (Homburg) or 18.0 cm (Mainz), and the slice thickness was 3.0 mm (Homburg) or 4.0 mm (Mainz). PET reconstruction was performed iteratively using a three-dimensional ordered-subset expectation maximization algorithm with three (Homburg) or two (Mainz) iterations, 24 (Homburg) or 33 (Mainz) subsets, Gaussian filtering, and 5.0 mm (Homburg) or 4.2 mm (Mainz) transaxial resolution at full-width at half maximum. CT was acquired for attenuation correction and anatomical localization using a low-dose scan. Aside from attenuation correction, decay correction, random correction, and scatter correction were performed. At each center, the PET/CT images were visually assessed by consensus of two board-certified nuclear medicine specialists, both of whom had extensive experience in interpreting ^68^Ga-PSMA-11 PET/CT images. ^68^Ga-PSMA-11 was produced in-house using PSMA-11 supplied by ABX advanced biochemical compounds GmbH (Radeberg, Germany) and ^68^Ga from a 68-germanium/^68^Ga generator (Eckert & Ziegler Strahlen- und Medizintechnik AG, Berlin, Germany). The synthesis of ^68^Ga-PSMA-11 was performed according to published procedures [28]. Quality control of the final product followed the rules of Good Manufacturing Practice (cGMP) guidelines. Radiopharmaceutical purity of >98% was required and tested by HPLC and iTLC.

### 2.3. Statistics

Data on patient and imaging characteristics and scan findings are reported as descriptive statistics, as applicable. Statistical tests were performed on Prism version 8 (GraphPad Software, San Diego, CA, USA). *p* values < 0.05 were deemed to be statistically significant. The chi-square test was used to compare overall suspicious lesion detection rates among PSA, PSA doubling time, and Gleason score subgroups as well as between study centers. Differences in SUV depending on lesion category were compared using the Mann–Whitney U test.

## 3. Results

**Figure 1:**
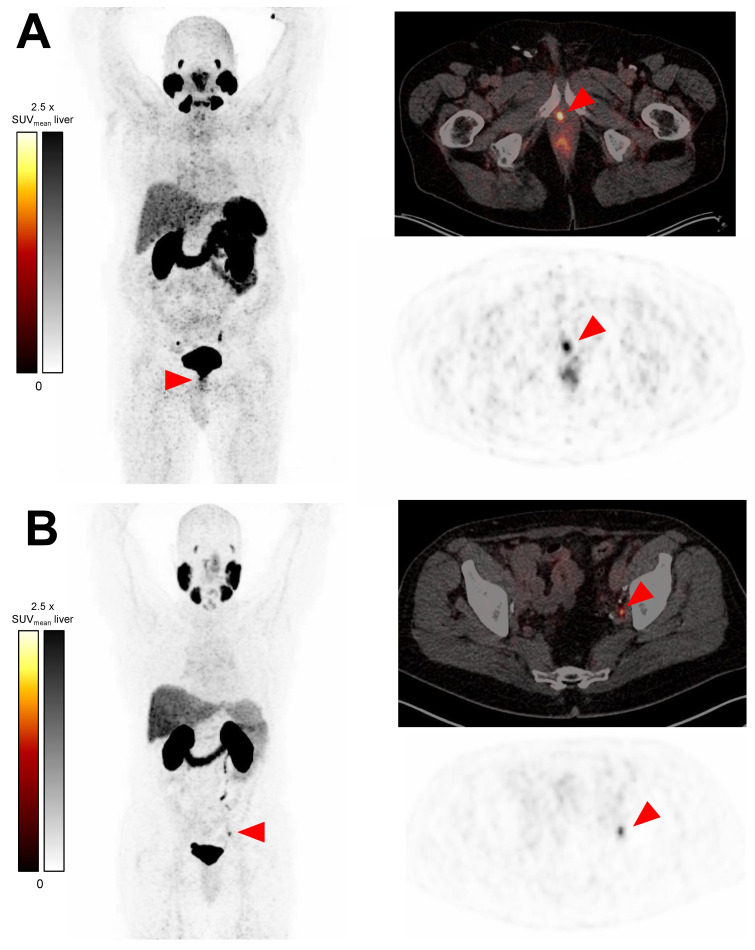
Representative ^68^Ga-PSMA-11 PET/CT transversal slice PET images (upper right-hand scans) and PET/CT fusion images (left-hand and lower right-hand scans) of suspicious lesions: (**A**) putative local recurrence in a 71-year-old man with a PSA concentration of 0.10 ng/mL and (**B**) a putative left iliac lymph node metastasis in a 67-year-old man with a PSA concentration of 0.13 ng/mL.

**Figure 2:**
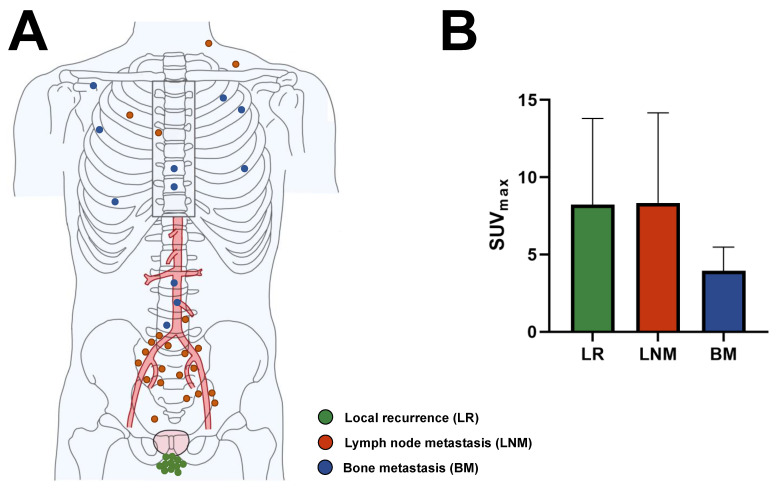
Characteristics of lesions suspicious for prostate cancer on ^68^Ga-PSMA-11 PET/CT in men with BCR and very low PSA concentration (N = 115). (**A**) Anatomic location and putative category of lesions (*n* = 44). Dots represent a single lesion and are color-coded as to putative category. (**B**) Descriptive statistics of SUV_max_ by suspicious lesion category. SUV_max_ does not differ (*p* = 0.87) between presumed local recurrences (*n* = 11) and lymph node metastases (*n* = 22) but is significantly lower (*p* < 0.01) for putative bone metastases (*n* = 11) than for the other lesion categories.

Overall, 29/115 patients (25.2%) have lesions suspicious of prostate cancer on ^68^Ga-PSMA-11 (Table 2). The positive scans show 44 suspicious lesions altogether (median [minimum–maximum] 1 [1–4] per patient with positive scan). These include 11 putative local recurrences, 22 putative lymph node metastases, and 11 putative bone metastases. No lesions suspicious of visceral metastasis are detected. The lowest PSA value is 0.02 ng/mL for a positive scan and 0.03 ng/mL each for a scan with ≥2 suspicious lesions (9/115, 7.8%). Representative images of positive scans are seen in Figure 1, and the specific anatomic sites of all detected lesions are depicted in Figure 2A.

The SUV_max_ is similar (*p* = 0.87) between putative local recurrences and presumed lymph node metastases. However, the SUV_max_ is significantly lower (*p* < 0.01) for bone metastases than for the other two lesion classifications (Figure 2B, Table 2).

**Figure 3:**
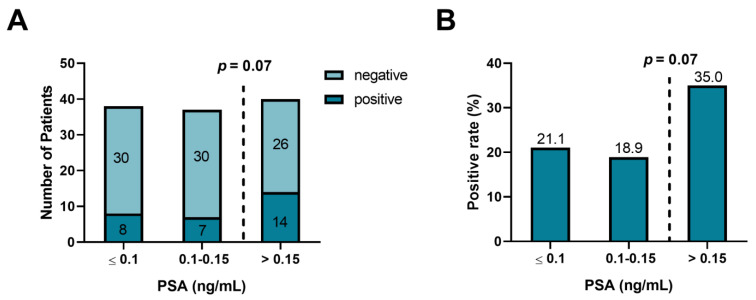
Detection rate on ^68^Ga-PSMA-PET/CT of lesions suspicious for prostate cancer by PSA level subgroup in 115 men with BCR and very low serum PSA concentration. Panel A depicts the numbers of patients with scans positive or negative for suspicious lesions, while Panel B provides detection rates in percent (numbers atop the bars). Patients with PSA > 0.15–0.2 ng/mL have a substantially higher detection rate than their counterparts with PSA ≤ 0.15 ng/mL, but the difference does not reach statistical significance (*p* = 0.07).

Figure 3 illustrates overall detection rates according to categories of PSA concentration. The PSA detection rates are 21.1% (8/38 patients), 18.9% (7/37 patients), and 35.0% (14/40 patients) for PSA levels < 0.1 ng/mL, 0.1–0.15 ng/mL and >0.15 ng/mL, respectively. The rate is markedly higher in patients with PSA > 0.15 ng/mL than in their counterparts with lesser PSA concentrations, but the difference only shows a possible trend toward statistical significance (*p* = 0.07).

**Table 2:** ^68^Ga-PSMA-11 PET/CT findings in 115 men with BCR and very low PSA concentration.

Variable	Value
Overall scan classification, % (n)	
Positive	25.2% (29)
Negative	74.8% (86)
Suspicious lesions on positive scans	
Total	44
Median (minimum–maximum)	1 (1–4)
Number of lesions/positive scan, % (n)	
1	69% (20/29)
2	14% (4/29)
3	14% (4/29)
4	3% (1/29)
Type of lesions, % (n)	
Local recurrence	25% (11/44)
Lymph node metastasis	50% (22/44)
Bone metastasis	25% (11/44)
Types of lesions on a positive scan, % (n)	
Local recurrence only	24% (7/29)
Lymph node metastasis only	34% (10/29)
Bone metastasis only	24% (7/29)
Local recurrence + lymph node	
metastasis	10% (3/29)
Local recurrence + bone metastasis	3% (1/29)
Lymph node metastasis + bone	
metastasis	3% (1/29)
SUVmax of suspicious lesions	
All suspicious lesions	
Median (minimum–maximum)	5.3 (2.1–26.3)
Mean ± SD	7.2 ± 5.3
Local recurrence	
Median (minimum–maximum)	5.7 (2.5–19.4)
Mean ± SD	8.2 ± 5.6
Lymph node metastasis	
Median (minimum–maximum)	6.6 (2.1–26.3)
Mean ± SD	8.3 ± 5.8
Bone metastasis	
Median (minimum–maximum)	3.3 (2.3–7.7)
Mean ± SD	3.9 ± 1.5

**Figure 4:**
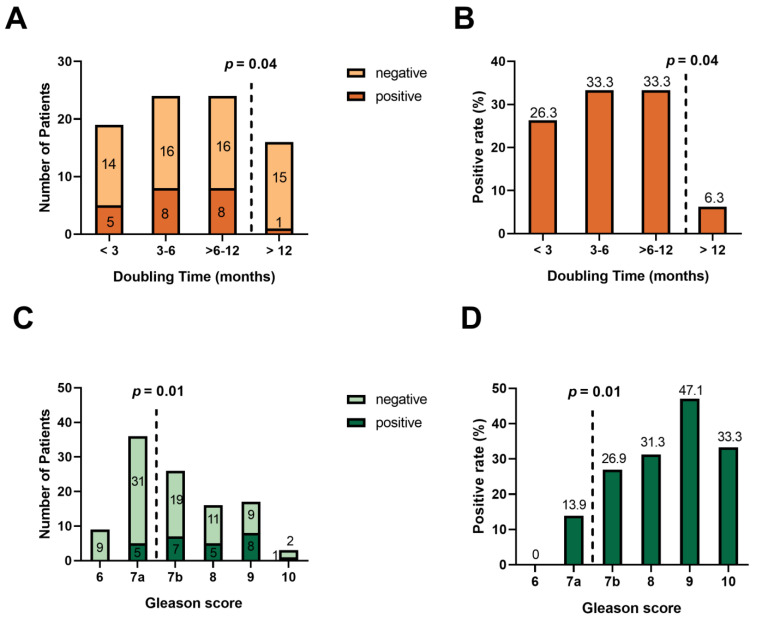
Rate of detection on ^68^Ga-PSMA-11 PET/CT of lesions suspicious for prostate cancer in 115 men with BCR and very low PSA concentration (**A**,**B**) by PSA doubling time category (in months) (*n* = 83) and (**C**,**D**) by Gleason score of surgical specimen (*n* = 107). Panels (**A**,**C**) show numbers of patients with positive or negative scans, while panels (**B**,**D**) depict the overall suspicious lesion detection rate in percent (numbers atop the bars). Detection rates are significantly higher (*p* = 0.04) in patients with doubling time < 12 months (*n* = 67) than in their counterparts with doubling time > 12 months (*n* = 16). Additionally, detection rates are significantly higher (*p* = 0.01) in men with a Gleason score ≥ 7b (*n* = 62) than in those with a Gleason score of 6 or 7a (*n* = 45).

Percentages represent proportions of the overall study sample or the subgroup with available data regarding a variable, as applicable. Due to rounding, not all percentages may add up to exactly 100%. Abbreviations: ^68^Ga, gallium-68; PSMA, prostate-specific membrane antigen; SD, standard deviation; and SUV_max_, maximum standardized uptake value. ^a^ Lesion classifications are putative and not biopsy-confirmed. Figure 4 shows overall detection rates according to PSA doubling time categories (*n* = 83) (Panels A–B) and Gleason score categories (*n* = 107) (Panels C–D). Scan positivity is significantly higher (*p* = 0.04) among men with doubling times ≤ 12 months than among those with slower doubling times. Indeed, only 1/16 patients (6%) in the latter subgroup have positive ^68^Ga-PSMA-11 PET/CT. The overall suspicious lesion detection rate is also significantly higher (*p* = 0.01) in men with Gleason scores ≥ 7b than in patients with lower Gleason scores. Of note, there is no significant difference in the positivity rate between the study centers (*p* = 0.24).

## 4. Discussion

The present analysis provides, from everyday practice over a long term in a large cohort, further evidence of the value of ^68^Ga-PSMA-11 PET/CT in localizing lesions suspicious for prostate cancer in prostatectomized patients with BCR but very low PSA levels. All of our patients have PSA concentrations at or below the European Association of Urology threshold to define BCR, 0.2 ng/mL [29], which is also the recommended level above PSMA where PET/CT should be performed, according to the current joint SNM and EANM guideline [30]. Notwithstanding those ultrasensitive PSA values and notwithstanding the well-established PSA concentration-dependence of ^68^Ga-PSMA-11 PET/CT [13,14], our per-patient scan positivity rate is 25.2% (29/115) by visual assessment. Our findings include multiple cases of one or more of not only putative local (prostate bed) recurrence but presumed lymph node and/or bone metastasis. Of interest, scan positivity is noted at PSA levels as low as 0.02 ng/mL and putative oligometastasis and/or bone metastasis at levels as low as 0.03 ng/mL. 

These detection rates are broadly in line with the 33% pooled rate reported for ^68^Ga-PSMA-11 PET/CT in 197 prostatectomized patients with PSA ≤ 0.19 ng/mL in the meta-analysis of Perera et al. [13] but are substantially lower compared with the 43.4% detection rate (98/226) reported by Afshar-Oromieh et al. [15] in the largest published cohort of prostatectomized men with PSA ≤ 0.2 ng/mL. In contrast, our scan positivity rates in the very low PSA BCR setting were a bit over twice those of Meredith et al. [16] in patients with PSA < 0.2 ng/mL, 11.3% (14/124). This discrepancy may be attributable to those investigators using a rather strict requirement for positivity, i.e., focal ^68^Ga-PSMA-11 uptake had to correspond with suspicious CT findings that we did not apply.

Unsurprisingly, the detection rate in our cohort is lower than those reported elsewhere in patients with higher PSA concentrations, e.g., 46% for 0.2–0.49 ng/mL, 57% for 0.5–0.99 ng/mL, 82% for 1–1.99 ng/mL, and 97% for ≥2 ng/mL in the Perera et al. meta-analysis [13]. However, analogous PSA concentration dependence of detection rates also appears to manifest within the very low PSA setting in our study. Scan positivity occurs 66% and 85% more frequently in our patients with PSA > 0.15 ng/mL than in their counterparts with PSA < 0.1 ng/mL or 0.1–0.15 ng/mL, respectively, albeit these differences approach but do not reach statistical significance (*p* = 0.07). This *p*-value was perhaps due to the relatively small size of the subgroups.

In our cohort, faster PSA doubling time and higher Gleason scores were associated with a higher rate of scan positivity. Comparable observations regarding the Gleason score appear in the literature [15,31].

Localization of prostate cancer recurrence may be expected to allow the selection of potentially less toxic and more effective fields and doses of radiation therapy. Such imaging results also could facilitate promising metastasis-directed approaches [32,33,34,35] in cases of oligometastatic disease. Given these potential benefits related to ^68^Ga-PSMA-11 PET/CT positivity, our results suggest that this modality may be considered even in the very low PSA BCR setting, especially when PSA doubling time is <12 months and/or the Gleason score is >7a.

When such scanning is negative, the reason(s) could be the short-lived ^68^Ga radionuclide (half-life ~68 min) precluding late acquisition of interpretable images, coupled with the presence of lesions internalizing the PSMA-targeted tracer unusually slowly and/or with persistent residual physiological activity due to protracted clearance of the radiopharmaceutical [36,37]. Preliminary data have suggested that imaging ≥24 h post-injection with PET tracers labeled with longer-lived radionuclides, such as zirconium-89 (half-life ~3.3 days), may frequently detect lesions missed by PET/CT using radiotracers labeled with ^68^Ga or other short-lived radionuclides, such as ^18^F (half-life ~110 min) [36,37,38,39,40].

The strengths and limitations of this analysis should be considered. The former includes a large study sample, dual-center participation, and long-term (~7 years) data from “real-world” practice, all contributing to the generalizability of our findings. Additionally, the statistically similar detection rates for the two centers suggest consistency in our results.

Among the downsides of the analysis were a retrospective study design, limited data on PSA doubling time, and above all, a lack of histological, clinical, or imaging follow-up data regarding suspicious lesions. These limitations decrease certainty as to whether the detected lesions indeed corresponded to prostate cancer. Nonetheless, the lesions’ locations, the considerable experience of the scan readers in acquiring and interpreting PET/CT images of men with prostate cancer, and the well-established sensitivity and specificity of ^68^Ga-PSMA-11 PET/CT to detect prostate malignancy [13] all lend plausibility to the lesions indeed comprising such tumor.

Notably, our study focused only on men who had undergone radical prostatectomy as primary treatment and on a single PSMA-targeted radiotracer, ^68^Ga-PSMA-11. It will be of interest to perform analogous analyses in men receiving radiation therapy as primary treatment and/or undergoing PET/CT with other such radiopharmaceuticals, e.g., ^18^F-PSMA-1007, ^18^F-DCFPyL, or ^68^Ga-PSMA-I&T. Especially the detection rate of local recurrence, which may be higher by using ^18^F-PSMA-1007 due to the hepatobiliary excretion mechanism with almost complete absence of urinary activity.

## 5. Conclusions

This was a retrospective analysis of a ~7-year, dual-center, “real world” experience with ^68^Ga-PSMA-11 PET/CT in a large group (*n* = 115) of post-prostatectomy patients with BCR and increasing but very low PSA levels of ≤0.2 ng/mL. Even in this setting, such imaging demonstrates a 25.2% (29/115) patient-level detection rate of lesions suspicious of prostate cancer. Given the increasingly clear patient outcome benefits of accurately localizing prostate cancer lesions at the earliest stage of recurrence, our observations suggest a potential value of ^68^Ga-PSMA-11 PET/CT in the very low PSA BCR setting, especially in cases with more rapid PSA doubling time or with high-risk histology.

## Figures and Tables

**Table 1:** Patient characteristics of 115 men with BCR and very low PSA concentration.

Characteristic	Value
Age, yr	
Median (minimum–maximum)	67.6 (48.4–85.7)
Mean ± SD	66.9 ± 8.0
PSA, ng/mL	
Median (minimum–maximum)	0.13 (0.01–0.2)
Mean ± SD	0.13 ± 0.06
Category, ng/mL, % (n)	
≤0.1	33.0% (38)
>0.1–0.15	32.2% (37)
>0.15–0.2	34.8% (40)
PSA doubling time, months	n = 83
Median (minimum–maximum)	4.9 (0.75–24)
Category, % (n)	
<3	23% (19)
3–6	29% (24)
>6–12	29% (24)
>12	19% (16)
Gleason score, % (n) unless otherwise noted	n = 107
Minimum–maximum	6–10
6	8.4% (9)
7a	33.6% (36)
7b	24.3% (26)
8	15.0% (16)
9	15.9% (17)
10	2.8% (3)
Additional treatment besides radical prostatectomy, % (n)	
Radiation therapy	8.7% (10)
Androgen deprivation therapy	8.7% (10)

Percentages represent proportions of the overall study sample or the subgroup with available data regarding a variable, as applicable. Due to rounding, not all percentages may add up to exactly 100%. Abbreviations: PSA, prostate-specific antigen, and SD, standard deviation.

## Data Availability

The datasets used and analyzed during the present study are available from the corresponding author upon reasonable request.

## Abbreviations

^18^F	Fluorine-18
^68^Ga	Gallium-68
BCR	Biochemical recurrence of prostate cancer
BM	Bone metastasis
LR	Local recurrence
LN	Lymph node metastasis
MIP	Maximum intensity projection
NA	Not available
PET/CT	Positron emission tomography/computed tomography
p.i.	Post-injection
PSA	Prostate-specific antigen
PSMA	Prostate-specific membrane antigen
SD	Standard deviation
